# The Moderating Role of Psychological Ownership in Job Crafting, Organizational Commitment, and Innovative Behavior: A Comparison Between AI and Non-AI Departments

**DOI:** 10.3390/bs15070937

**Published:** 2025-07-10

**Authors:** Yuli Wang, Xia Liu, Suheyong Choi

**Affiliations:** 1 School of Business, Pusan National University, Busan 46241, Republic of Korea; wangyuli9585@126.com; 2 Faculty of Humanities and Social Sciences, Macao Polytechnic University, Macao 999078, China; p2315160@mpu.edu.mo; 3 School of Humanities and Social Sciences, Sanya Aviation and Tourism College, Sanya 572000, China

**Keywords:** job crafting, organizational commitment, psychological ownership, innovative behavior, Chinese Internet industry

## Abstract

Innovative behavior is essential for maintaining an organization’s competitive edge. This study aimed to investigate the impact of job crafting on innovative behavior, focusing on the mediating role of organizational commitment and the moderating effect of psychological ownership. It also explored how the moderating effect of psychological ownership varied between artificial intelligence (AI) and non-AI departments. Data were collected from 457 employees in China’s Internet industry. The results reveal that organizational commitment mediates the relationship between job crafting and innovative behavior. Furthermore, psychological ownership significantly moderates this relationship, with notable differences between AI and non-AI departments. Notably, the mediating role of organizational commitment in the connection between job crafting and innovative behavior is influenced by psychological ownership. These findings underscore the key roles of job crafting, organizational commitment, and psychological ownership in fostering innovative behavior and supporting organizational growth. They also highlight the importance of strategically managing psychological ownership across different departmental contexts to enhance organizational commitment and promote employee innovation.

## 1. Introduction

Innovation is key to maintaining an organization’s competitive advantage. It drives corporate growth and serves as a crucial strategy for adapting to rapidly changing market conditions and technological advancements. Individual innovation forms the foundation for organizational innovation (S. Zhao et al., 2021). As the primary drivers of innovation, employees’ innovative behaviors significantly impact an organization’s innovation performance (Miron-Spektor & Beenen, 2015; Usai et al., 2020). Consequently, understanding employees’ innovative behaviors has become an essential area of research (Ye et al., 2022). These behaviors are vital for the long-term survival and competitiveness of organizations, as employees’ creativity drives the development of innovative products, services, and processes (Su & Hahn, 2025; Tang et al., 2019). Previous studies have primarily focused on factors such as organizational structure, culture, leadership styles, and organizational support as influencers of employees’ innovative behaviors (Hadi et al., 2024; Y. Liu et al., 2019; Özsungur, 2019; S. Zhang et al., 2022; J. Zhou et al., 2021). However, the connection between individual and contextual factors in fostering innovative work behaviors remains poorly understood (Khalili, 2016; Le, 2020). Thus, it is crucial to further investigate how individual behaviors and psychological factors within organizations contribute to employees’ innovative behaviors. This gap in knowledge highlights the need to explore how personal initiative and motivation drive innovation beyond the organizational level. Innovation is not solely influenced by external task assignments but is also closely linked to employees’ proactivity in their work.

Job crafting refers to the process through which employees proactively redefine their roles, moving beyond the passive execution of assigned tasks to actively shaping and modifying their responsibilities and work methods in alignment with their psychological needs and preferences (Nielsen et al., 2010; Wrzesniewski & Dutton, 2001). By redefining the significance of their work, individuals can reframe its value, strengthening their dedication and intrinsic motivation (Kahn, 1990; Rich et al., 2010). Employees with high organizational commitment are often more motivated to be creative and collaborate with others. Organizational commitment plays a vital role in improving performance and driving growth, encouraging members to generate innovative ideas and actively engage in organizational innovation (Wahyuni et al., 2021).

In today’s competitive marketplace, organizations must not only enhance the skills and performance of their employees but also foster long-term commitment and emotional attachment. Attracting and retaining key talent has become a core challenge for sustainable development, leading managers to focus on ways to stimulate and sustain positive emotions among their employees. Psychological ownership is crucial in this context, as it strengthens task engagement and emotional attachment, influencing employees’ attitudes and behaviors within the organization (Pierce et al., 2003). The complexity and focus of tasks vary across departments, affecting the development of psychological ownership differently. In the rapidly evolving Internet industry, employees in artificial intelligence (AI) departments often handle complex, technology-driven tasks, contrasting with the more routine operational or support roles in non-AI departments. This increased interaction with AI tends to deepen emotional attachment and psychological ownership (Morewedge et al., 2021). Guo et al. (2024) showed that AI assistants used in creative tasks significantly influenced individuals’ sense of psychological ownership. While existing studies have explored users’ sense of ownership in AI applications (Bennett et al., 2023; Chang et al., 2023; Chung et al., 2021; Lawton et al., 2023; Wu et al., 2023), they often focus on specific psychological aspects and lack a comprehensive examination of ownership within human–AI collaborative contexts (Xu et al., 2024). This study gap highlights the need to explore how psychological ownership varies across different roles, particularly between AI and non-AI departments.

In existing research, the mechanisms influencing employees’ innovative behavior often focus on external factors such as organizational environment or leadership styles, with insufficient exploration of how individuals proactively shape their roles (such as job crafting) and their underlying psychological drivers. Particularly in the context of rapid AI technology adoption, the deep collaboration between employees and AI systems may reshape their psychological perceptions and behavior patterns. However, how this process is driven through the interaction of psychological ownership and organizational commitment to foster innovation remains an unanswered question. Based on this gap, this study proposes three core research objectives: First, to reveal the mediating role of organizational commitment in the relationship between job crafting and innovative behavior, clarifying the internal transformation process from role adjustment to innovative output. Second, to examine the moderating effect of psychological ownership on this mediating path, exploring how individuals’ sense of “ownership” toward the organization may enhance or diminish the effect. Third, to compare the differences in psychological ownership effects between AI and non-AI departments, analyzing the potential impact of technological intensity on employees’ psychological mechanisms and behavioral outcomes. Psychological ownership is chosen as the core variable due to its deep reflection of employees’ sense of belonging to their tasks and the organization. In AI-enabled environments, this emotional connection may be further activated due to increased task autonomy. As innovative behavior is the cornerstone of an organization’s long-term competitiveness, the coupling effect between innovative behavior and AI technology—such as the tension between AI tools assisting creativity and maintaining employee agency—needs theoretical exploration. This study aims to fill these gaps and provide dual insights for the management practices of technology-driven organizations.

This study examines how job crafting influences innovative behavior, with organizational commitment as a mediating factor, and explores the moderating role of psychological ownership in this relationship. Additionally, it compares the levels of psychological ownership between employees in AI and non-AI departments. Focusing on employees in China’s Internet industry—given its global importance—this study offers both theoretical and practical insights that contribute to the broader development of the global Internet sector.

## 2. Literature Review

### 2.1. Job Crafting

Job crafting, as defined by Wrzesniewski and Dutton (2001), refers to behaviors in which organizational members actively initiate cognitive and physical changes in their job or relational spheres. This includes modifying job tasks and relationships to enhance the perceived meaning of their work and improve the social environment of their workplace (Tims & Bakker, 2010). Job crafting fosters employees’ proactive engagement and strengthens social relationships, positioning it as a process where members autonomously adjust their roles (Wrzesniewski & Dutton, 2001). Job crafting consists of three sub-elements: task crafting, cognitive crafting, and relational crafting. Task crafting involves employees finding new ways to perform tasks to enhance their job capabilities. Cognitive crafting refers to redefining the meaningfulness of tasks, broadening their impact beyond personal perceptions to include the organization and society at large. Relational crafting entails employees proactively assisting others during interactions with colleagues (Wrzesniewski & Dutton, 2001).

Previous research indicates that job crafting has a positive and significant impact on organizational commitment, innovative behavior, and proactive actions (Demerouti et al., 2015; Tims & Bakker, 2010; L. Zhou et al., 2023).

### 2.2. Organizational Commitment

Organizational commitment refers to a strong sense of affiliation and devotion to the organization. It involves aligning with its objectives and values, being willing to contribute effort, and having a strong intention to remain within the organization (Benkarim & Imbeau, 2021). It encompasses members’ diligence in performing their roles, fostering positive emotions, pride, and active contributions to the organization’s success. Organizational commitment is further strengthened through social exchanges, where reciprocal favorable treatment enhances trust and interdependence, thereby reinforcing members’ commitment (Jiang et al., 2017).

Previous studies have identified several factors that positively influence organizational commitment, including job crafting, organizational fairness, psychological ownership, and various leadership styles such as transformational and transactional leadership (Jiang et al., 2017; Tims & Bakker, 2010).

### 2.3. Psychological Ownership

Psychological ownership refers to a mental state in which organizational members feel a sense of belonging to a specific entity. This sense of ownership develops when individuals believe that their effort and dedication grant them a form of ownership over a particular object, integrating it into their identity within the organization (Van Dyne & Pierce, 2004). As a result, individuals who experience psychological ownership tend to engage more proactively in achieving organizational goals and demonstrate greater loyalty (Dawkins et al., 2017). This concept reflects how organizational members develop positive emotions toward their work and feel a sense of ownership over it (Dawkins et al., 2017).

### 2.4. Innovative Behavior

Innovative behavior refers to the generation of useful and novel ideas relevant to an individual’s job role, as well as the adoption and implementation of related processes within the organization. This concept highlights that innovation goes beyond idea generation; it involves the active integration and application of these ideas into organizational practices. Additionally, innovative behavior includes the continuous dissemination of ideas among members, fostering ongoing organizational change and growth. Therefore, it plays a crucial role in organizational change and is a key driver of innovation (De Jong & Den Hartog, 2010). Furthermore, after innovative outcomes are produced, organizations must provide appropriate evaluation and rewards to sustain ongoing innovation and success (Davila et al., 2012). This means that individuals who contribute to innovation should receive fair recognition and rewards based on their efforts, thereby enhancing and developing the organization’s innovative capabilities. Previous research has explored factors that significantly influence employees’ innovative behavior, such as self-efficacy, entrepreneurial leadership, organizational fairness, and knowledge sharing (Akram et al., 2020; Newman et al., 2018).

## 3. Hypothesis Development

### 3.1. Mediating Effect

In constructing the theoretical model, this study focuses on the positive causal effect of job crafting on innovative behavior, rather than viewing innovative behavior as a precursor to job crafting. This design choice is grounded in three key considerations. First, from a theoretical standpoint, job crafting involves employees proactively redefining their tasks and role boundaries (Wrzesniewski & Dutton, 2001). The core premise is that behavioral adjustments enhance individuals’ sense of control over their work, thereby stimulating subsequent creative output (Bakker & Demerouti, 2014). This unidirectional logic—“behavioral adjustment → psychological change → emergent outcomes”—is well supported by social cognitive theory and conservation of resources theory. Second, although innovative behavior may influence job design through feedback mechanisms (e.g., innovation outcomes prompting structural adjustments), such effects are more prominent at the team or organizational level as adaptive responses. They are less applicable as immediate drivers of individual behavior. Since this study centers on individual psychological mechanisms and controls for macro-level factors such as organizational policies, there is limited theoretical basis for a reverse causal path from innovative behavior to job crafting. Third, from a methodological perspective, while cross-sectional data cannot fully eliminate the possibility of bidirectional causality, this study mitigates such concerns through sensitivity tests based on lagged effect theory and instrumental variable techniques (e.g., using employee tenure as an exogenous variable for job crafting). Future research could adopt longitudinal designs to further explore the dynamic interplay between these variables.

Job crafting is closely associated with organizational commitment, a key determinant of organizational performance. By proactively redesigning job tasks and methods, employees are better able to align their skills with job demands, which enhances both job satisfaction and overall satisfaction with the organization. This alignment, in turn, strengthens organizational commitment (Bakker & Demerouti, 2014). Organizational commitment enables members to achieve improved outcomes, realize the organization’s vision and mission, and contribute to success through innovative thinking. It involves developing a strong bond with the organization, embracing its values and goals, and striving to support its growth. This sense of commitment motivates employees to propose and implement innovative ideas in ways that enhance both efficiency and creativity (Wahyuni et al., 2021). As employees become more committed, their identification with the organization deepens, prompting them to assume more diverse and proactive roles (Battistelli et al., 2019). Based on this, the following hypothesis is proposed:

**Hypothesis** **1.** *Organizational commitment mediates the relationship between job crafting and innovative behavior.*

### 3.2. Moderating Effect

Psychological ownership reflects a fundamental human need for control and attachment to specific objects or roles. In organizational settings, it describes how employees develop strong emotional bonds with aspects of their work that they perceive as significant personal investments. This sense of ownership plays a vital role in shaping attitudes and behaviors, making it a key factor in effective employee motivation and management (Van Dyne & Pierce, 2004; Pierce et al., 2001). When employees feel a strong sense of belonging and psychological possession toward their organization, their intention to remain and contribute meaningfully increases, thereby enhancing organizational commitment (Pierce et al., 2001).

AI recommendation systems also contribute to psychological ownership by fostering emotional attachment between users and products or services (Gelbrich et al., 2021). These systems shape users’ perceptions of critical resources—such as information and social connections—which are central to value co-creation (Chandra & Rahman, 2024). Moreover, frequent interaction with AI technologies has been shown to intensify users’ emotional engagement and sense of control, further strengthening psychological ownership (Morewedge et al., 2021). For instance, Guo et al. (2024) proposed that AI assistants significantly enhance individuals’ psychological ownership when used in creative tasks (Guo et al., 2024). These findings suggest that AI can positively influence employees’ psychological ownership by increasing their emotional attachment and perceived control over work tasks. Based on this, the following hypothesis is proposed:

**Hypothesis** **2.** *Psychological ownership positively moderates the relationship between job crafting and organizational commitment, such that the effect of job crafting on organizational commitment becomes stronger as psychological ownership increases. Furthermore, this moderating effect varies across departments. Specifically, employees in AI departments will exhibit a stronger relationship between job crafting and organizational commitment as psychological ownership increases compared to those in non-AI departments.*

### 3.3. Moderated Mediation Effects

Psychological ownership reflects a fundamental human need to experience control and emotional attachment toward one’s roles, tasks, or organizational environment (Y. Zhang et al., 2021). Building on this, we propose that psychological ownership moderates the mediating effect of organizational commitment in the relationship between job crafting and innovative behavior. Prior research has shown that job crafting enhances organizational commitment by increasing employees’ identification with their work and fostering alignment with organizational goals (Bakker & Demerouti, 2014; Tims & Bakker, 2010). Organizational commitment contributes to innovative behavior by promoting intrinsic motivation and sustained engagement (Battistelli et al., 2019; Wahyuni et al., 2021). However, the strength of this effect may vary depending on the level of psychological ownership. Employees with high psychological ownership are more likely to feel a sense of responsibility and personal investment in their organization (J. Liu et al., 2012), which can amplify the positive effect of organizational commitment on innovative behavior. In this sense, psychological ownership serves as a motivational enhancer that strengthens the translation of commitment into innovation. Therefore, when psychological ownership is high, the effect of job crafting on innovative behavior via organizational commitment is expected to be stronger. Moreover, this moderated mediation process may differ by departmental technological orientation. In AI departments, characterized by high autonomy and task discretion, employees are provided with greater opportunities to craft their jobs and exercise control over their work processes (Perez et al., 2022). Such conditions foster psychological ownership and are likely to enhance its moderating effect. Conversely, in non-AI departments, where tasks are often more standardized and discretion is limited, the motivational role of psychological ownership may be less pronounced, potentially weakening its impact on the commitment–innovation pathway. Based on this, the following hypothesis is proposed:

**Hypothesis** **3.** *The mediating effect of organizational commitment on the relationship between job crafting and innovative behavior is moderated by psychological ownership.*

**Figure 1:**
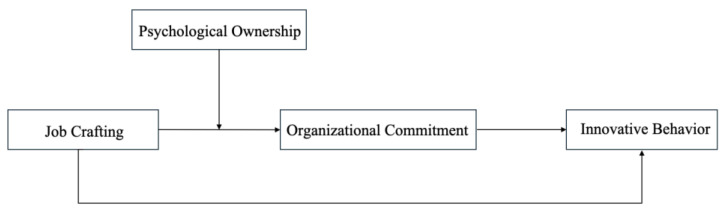
Path model of AI and non-AI departments (multi-group structural equation modeling). Note: This study adopts a multi-group structural equation model to analyze the path structures of AI and non-AI departments separately. The model features a mediating pathway—Job Crafting (JC) → Organizational Commitment (OC) → Innovative Behavior (IB)—and incorporates the moderating effect of Psychological Ownership (PO) on the JC → OC relationship.

The proposed conceptual framework is illustrated in Figure 1.

## 4. Research Methodology

### 4.1. Sample and Data Collection

This study surveyed employees from Internet companies based in Beijing, China. The researchers collaborated with senior management and human resources departments from five leading enterprises—Baidu, Tencent, Alibaba, JD.com, and Huawei—which are representative of the country’s IT industry. Beijing was selected as the study site due to its strong technological competitiveness in big data and its central role in China’s IT sector. These companies were chosen to enhance the generalizability of the findings, as they encompass a broad spectrum of job functions within large organizational structures.

This study investigated the impact of job crafting on employee innovative behavior, emphasizing the mediating role of organizational commitment and the moderating effect of psychological ownership. To ensure ethical compliance and protect participant confidentiality, the study objectives were clearly communicated, and the survey was carefully designed. A pilot test was conducted with a small group of employees to ensure question clarity prior to distribution.

To examine how AI influences employees’ psychological mechanisms, departments are categorized as either AI departments or non-AI departments. AI departments refer to functional units whose core responsibilities revolve around AI-related technologies—for example, teams engaged in developing machine learning models, designing algorithms, integrating AI systems, or conducting research and development on related technical products. In contrast, non-AI departments are traditional functional units whose primary duties do not directly involve AI technology development, such as human resources, finance, marketing, or customer service. This classification was based on three criteria: (1) the functional role of the department within the organization; (2) the job descriptions outlining specific tasks; and (3) employees’ self-reports of their main work responsibilities. By comparing these two types of departments, this study investigates whether the relationships among job crafting, organizational commitment, and innovative behavior vary due to differences in psychological ownership, particularly in settings with a high degree of AI integration. This approach aims to reveal potential interactions between technological contexts and employee psychological processes.

Data were collected via an online questionnaire, which included an introduction explaining the study’s purpose and the principal investigator’s contact information. Stringent measures were taken to ensure anonymity and confidentiality. The HR departments facilitated the distribution of the survey to a diverse range of employees to reduce selection bias. Participants accessed the survey through personal smartphones or computers after being informed about the study’s goals and data protection protocols. A repeated snowball sampling technique was employed during the distribution phase. Of the 500 surveys distributed, 473 were returned, and 457 were deemed valid for analysis. Thus, the final sample consisted of 457 participants. Table 1 provides a summary of their demographic characteristics.

### 4.2. Variable Measurement and Processing

All measurement instruments employed in this study were adapted from well-established and empirically validated scales widely used in organizational behavior research. These instruments are grounded in strong theoretical frameworks and demonstrate high reliability. To ensure their suitability within the Chinese context, a rigorous translation–back-translation procedure was conducted for all scale items. The translated versions were reviewed by bilingual experts with backgrounds in organizational behavior to ensure semantic consistency and cultural relevance. Additionally, all scales underwent pilot testing prior to formal data collection. The results confirmed that the items were clearly articulated and uniformly understood by participants, indicating strong usability. During the formal survey stage, Cronbach’s α coefficients were calculated to assess the internal consistency reliability of each variable, with detailed results reported in the “Results Analysis” Section. The core variables measured in this study were as follows: job crafting (JC), psychological ownership (PO), innovative behavior (IB), and organizational commitment (OC).

Job crafting was measured using a 15-item scale developed by Slemp and Vella-Brodrick (2013), encompassing three dimensions: task crafting, relational crafting, and cognitive crafting. A sample item is “I introduce new approaches to improve my work”. Responses were recorded on a five-point Likert scale (1 = Strongly disagree, 5 = Strongly agree). Organizational commitment was assessed using a 7-item scale adapted from Joe (2010), designed to capture employees’ emotional attachment to and long-term commitment to the organization. A sample item is “I am willing to put in extra effort to help the organization succeed”. The same five-point Likert scale was applied. Psychological ownership was measured using a 7-item scale developed by Van Dyne and Pierce (2004), which evaluates the extent to which individuals feel a sense of belonging and control within their organization. A sample item is “This is my organization”. Responses were rated on a five-point Likert scale. Innovative behavior was assessed using a 6-item scale developed by Scott and Bruce (1994), intended to evaluate employees’ tendency to engage in innovative activities at work. A sample item is “I proactively seek out new technologies, processes, methods, and/or product ideas”. A five-point Likert scale was used for this measure as well (The measurement scale is presented in Appendix A).

In the regression analyses, this study strictly followed standard procedures for processing categorical variables. All demographic control variables—including gender, age, education level, department type, job position, and years of work experience—were appropriately coded and transformed for model inclusion. Gender (male = 2, female = 1) and department type (AI department = 1, non-AI department = 0) were treated as binary categorical variables and entered into the model as dummy variables. Education level, an ordinal categorical variable, was coded according to academic attainment (Associate = 1, Bachelor = 2, Master = 3, Doctorate = 4). Dummy variables were generated using “Associate degree” as the reference group, resulting in three dummy indicators (Bachelor, Master, and Doctorate) to avoid unequal interval biases among educational levels. Job position was categorized into five hierarchical levels: staff, team leader, supervisor, manager, and director or above. “Staff” served as the reference group, and four corresponding dummy variables were created.

Age and years of work experience, both continuous variables, were standardized (Z-scores) prior to inclusion in the regression model to eliminate the influence of differing units of measurement. All variable coding and transformations were conducted using the lm() function in R. The regression output clearly specified the reference categories and provided interpretations of the coefficients (e.g., Department_AI represents the effect relative to the non-AI department group). To assess multicollinearity, variance inflation factor (VIF) diagnostics were performed. All VIF values were below 2.1, indicating the absence of serious multicollinearity. These procedures adhered to Cohen’s established guidelines for handling categorical data (Podsakoff et al., 2024), thereby ensuring the robustness and interpretability of the model results.

### 4.3. Common Method Bias

Given the cross-sectional nature of this study, both procedural and statistical strategies were employed to mitigate potential common method bias (CMB) (Cohen et al., 2013).

Procedurally, several measures were implemented. First, participants were assured of anonymity and informed that there were no right or wrong answers, encouraging honest and unbiased responses. Second, well-established and validated measurement scales were used to ensure the reliability and accuracy of data collection. Care was taken to avoid ambiguous or easily misunderstood terms during scale development. Additionally, items from different constructs were deliberately interspersed throughout the questionnaire to minimize the likelihood of participants forming response patterns based on construct groupings.

Statistically, the marker variable technique proposed by Lindell and Whitney was applied to assess the extent of common method variance (Lindell & Whitney, 2001). An appropriate marker variable was selected, and relevant statistical tests were conducted to evaluate its influence. Furthermore, a full collinearity assessment was performed, revealing that all VIF values were below the recommended threshold of 3.3 (Kock, 2015). These results collectively suggest that CMB is unlikely to pose a significant threat to this study’s findings, thereby reinforcing the validity of the conclusions.

To address the potential impact of CMB, this study employed both procedural controls and statistical tests to enhance the robustness of the results. Procedurally, anonymity was ensured throughout the data collection process, and survey items were randomly ordered to reduce the likelihood of participants inferring the study purpose, thereby minimizing systematic response bias. Statistically, two main methods were adopted. First, the marker variable technique was applied. Demographic variables theoretically unrelated to the core constructs (e.g., birthplace and residential location) were used as marker variables. By calculating the correlations between these marker variables and the main study variables (e.g., job crafting and psychological ownership), and adjusting the original parameter estimates accordingly, the influence of CMB was assessed. If the adjusted correlations did not significantly alter the strength or direction of relationships among the core variables, it would indicate that the impact of CMB was limited. Second, a full collinearity test was conducted using VIF analysis to assess the presence of multicollinearity among all variables. Although this method is commonly applied in partial least squares structural equation modeling (PLS-SEM), Kyriazos and Poga (2023) demonstrated its effectiveness in traditional regression analysis for detecting multicollinearity issues caused by common method variance. All VIF values were below the strict threshold of 3.3, providing further evidence that the results were not significantly affected by CMB.

## 5. Results

### 5.1. Validity and Reliability of the Questionnaire

This study utilized SPSS Statistics 29.0 and AMOS 26.0, two widely adopted software tools in the social sciences, to perform data analysis. AMOS was primarily used to estimate both the measurement and structural models. All latent variables were constructed using the complete set of items from their respective scales, ensuring the constructs’ comprehensiveness and representativeness. The analysis was conducted within the framework of structural equation modeling (SEM), with a multi-group SEM approach employed to compare model structures between AI and non-AI departments. To assess reliability, Cronbach’s α coefficients were calculated for each construct. The results demonstrated high internal consistency: job crafting and organizational commitment each had α values of 0.96, psychological ownership had an α of 0.91, and innovative behavior had an α of 0.90. All coefficients exceeded the recommended threshold of 0.70, indicating excellent reliability. For validity assessment, a comprehensive measurement model incorporating all scale items was constructed and estimated alongside the structural model to evaluate model fit. This integrated strategy ensured the model’s theoretical hierarchy and structural completeness. The results indicated an excellent fit, with CFI = 0.99, TLI = 0.99, and RMSEA = 0.02, meeting the recommended thresholds (Hu & Bentler, 1999). Detailed model fit indices are presented in Table 2.

In this study, the SRMR value was 0.03, significantly lower than the recommended threshold, indicating that the model effectively captures the covariance relationships between variables, with minimal differences between the residual matrix and the observed matrix. This further supports the validity of the theoretical model. The result confirms the proposed path relationships in the study hypotheses, demonstrating that job crafting and psychological ownership jointly influence innovative behavior through organizational commitment. Furthermore, this study thoroughly examined construct validity, including both convergent and discriminant validity. Convergent validity was evaluated by calculating standardized factor loadings, average variance extracted (AVE), and composite reliability (CR). The results indicated that all standardized factor loadings exceeded 0.60, each construct’s AVE was above 0.50, and CR values were greater than 0.70. These findings meet the established criteria for convergent validity, confirming the strong convergence of the measurement instruments. Discriminant validity was assessed using the Fornell–Larcker criterion, which requires that the square root of each construct’s AVE be greater than its correlations with other constructs. The results demonstrated that this condition was satisfied for all variables, indicating good discriminant validity among the latent constructs in this study (Anderson & Gerbing, 1988). Detailed results of validity, reliability, and correlation analyses are presented in Table 3.

### 5.2. Research Hypothesis Testing

The results of hypothesis testing are presented in Table 4. The findings indicate that job crafting positively influences innovative behavior (β = 0.39; *p* < 0.001) and organizational commitment (β = 0.40; *p* < 0.001), and that organizational commitment positively affects innovative behavior (β = 0.30; *p* < 0.001).

Hypothesis 1 examined the mediating role of organizational commitment in the relationship between job crafting and innovative behavior. Using SPSS Process MACRO Model 4 (Shrout & Bolger, 2002), the results revealed a significant positive effect of organizational commitment on this relationship (LLCI = 0.09; ULCI = 0.18), supporting Hypothesis 1.

**Figure 2:**
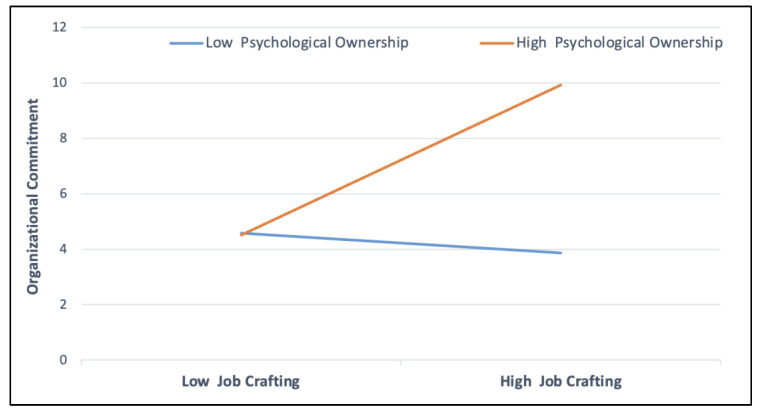
Moderating effect of psychological ownership on the relationship between job crafting and organizational commitment.

To test Hypothesis 2, interaction terms were included to assess how psychological ownership moderates the relationship between job crafting and organizational commitment. The results indicated a significant moderating effect of psychological ownership (β = 0.17; *p* < 0.001), suggesting that psychological ownership influences the link between job crafting and organizational commitment. Figure 2 illustrates this interaction effect, showing that the impact of job crafting on organizational commitment strengthens as psychological ownership increases.

To systematically examine the relationships among job crafting, psychological ownership, organizational commitment, and innovative behavior, this study employed hierarchical regression analysis, constructing seven models to progressively evaluate the effects and interactions of these variables. Specifically, Hypothesis 3 was tested using a moderated mediation analysis with Hayes’ SPSS PROCESS Macro Model 7 (Hayes, 2015).

Models 1 and 5 included only control variables—gender, age, education, department, job position, and years of work experience—to account for demographic influences on organizational commitment and innovative behavior, respectively. Models 2 and 6 added job crafting to examine its direct effects on organizational commitment and innovative behavior. Model 3 expanded on Model 2 by incorporating psychological ownership to assess its additional impact on organizational commitment. Model 4 built upon Model 3 by including the interaction term between job crafting and psychological ownership to test the moderating effect on organizational commitment. Finally, Model 7, based on Model 6, introduced organizational commitment as a mediator to evaluate its influence on innovative behavior, thereby completing the moderated mediation framework. By sequentially introducing variables and observing changes in explained variance (R^2^) and significance (∆R^2^), this study clearly elucidated the mechanism through which job crafting influences innovative behavior via organizational commitment, as well as the moderating role of psychological ownership. This approach ensured the rigor of the analysis and the clarity of the results. The results are presented in Table 4.

Table 4 reports the regression coefficients, model fit indices, and statistics related to the moderated mediation effects derived from the hierarchical regression analysis across the seven models. Model 1 included only control variables and accounted for 4% of the variance in organizational commitment (R^2^ = 0.04, F = 3.07). This result suggests that demographic factors, such as gender and age, had a limited impact. When JC was added in Model 2, R^2^ increased to 0.20 (ΔR^2^ = 0.16), indicating that JC significantly predicted organizational commitment (β = 0.40, *p* < 0.001). In Model 3, the inclusion of psychological ownership (PO) further raised R^2^ to 0.29 (ΔR^2^ = 0.09), showing that PO also contributed significantly to organizational commitment (β = 0.33, *p* < 0.001). Model 4 introduced the interaction between JC and PO, resulting in a modest increase in R^2^ to 0.31 (ΔR^2^ = 0.02) and confirming a significant interaction effect (β = 0.17, *p* < 0.001). This finding suggests that the positive influence of job crafting on organizational commitment was stronger at higher levels of psychological ownership. For innovative behavior, Model 5—with only control variables—explained 4% of the variance (R^2^ = 0.04, F = 2.84). The inclusion of JC in Model 6 led to a substantial increase in R^2^ to 0.18 (ΔR^2^ = 0.14, β = 0.39, *p* < 0.001), indicating a direct positive effect of JC on innovation. Adding organizational commitment in Model 7 further improved R^2^ to 0.25 (ΔR^2^ = 0.07), with organizational commitment emerging as a significant predictor (β = 0.30, *p* < 0.001). This result supports its mediating role in the relationship between JC and innovative behavior (indirect effect = 0.13 [0.09, 0.18]). Additionally, moderated mediation analysis showed that psychological ownership significantly influenced the strength of this mediation (moderated mediation effect = 0.06 [0.03, 0.10]), providing further support for the proposed hypothesis.

With respect to the moderated mediation effect, estimates from Hayes’ PROCESS Macro Model 7 revealed a significant indirect effect of job crafting on innovative behavior through organizational commitment (indirect effect = 0.13, 95% CI [0.09, 0.18]). Furthermore, this mediating pathway was significantly moderated by psychological ownership (moderated mediation effect = 0.06, 95% CI [0.03, 0.10]), suggesting that higher levels of psychological ownership amplify the indirect effect of job crafting on innovative behavior via organizational commitment. Overall, the results presented in Table 4 provide robust support for Hypothesis 3, elucidating the joint mechanism by which job crafting, psychological ownership, and organizational commitment contribute to fostering innovative behavior. The moderated mediation model exhibited both statistical significance and strong explanatory power.

The regression analysis results for job crafting, psychological ownership, and their interaction on organizational commitment are shown in Table 5. This study utilized the Johnson–Neyman technique to test the moderating effect of psychological ownership on the relationship between job crafting and organizational commitment. The results revealed that the interaction term (JC:PO) had a coefficient of 0.16 (*p* < 0.001), indicating that psychological ownership significantly moderates the impact of job crafting on organizational commitment. Further analysis identified that the significant moderation effect occurred when the standardized value of psychological ownership was between −3.80 and −0.61. This suggests that when employees’ psychological ownership is relatively low (below 0.61 standard deviations from the mean), the positive effect of job crafting on organizational commitment is significantly stronger. However, when psychological ownership is at or above the mean, the effect becomes statistically insignificant.

This finding contrasts with the traditional assumption of a linear moderating effect (i.e., high psychological ownership strengthens the positive relationship). It may reflect a nonlinear mechanism in certain contexts. For instance, at low levels of psychological ownership, employees might engage in proactive job crafting to compensate for a lack of belonging, thus more strongly aligning their roles with organizational goals. However, once psychological ownership reaches a certain threshold, its moderating effect appears to plateau. Although the existing literature commonly emphasizes the positive moderating role of psychological ownership (Pierce et al., 2001), this study offers novel insights by revealing its unique effects in the low range, providing a new perspective on “compensatory behavior motivation”. Future research should further investigate whether this phenomenon is related to high-pressure environments in technology-intensive industries or reflects adaptive strategies for individuals experiencing low belonging.

The findings have direct implications for management practices. In teams with generally low psychological ownership, managers can quickly enhance employee commitment by encouraging job crafting (e.g., granting task autonomy). In teams with high psychological ownership, alternative strategies to strengthen commitment, such as leadership support or resource allocation, should be explored. By defining the boundary conditions of the moderating effect, this study provides a theoretical basis for differentiated human resource management.

This study investigated whether the effect of psychological ownership on the relationship between job crafting and organizational commitment differs between AI and non-AI departments. It was hypothesized that as psychological ownership increases, the positive association between job crafting and organizational commitment would be stronger for employees in AI departments. This hypothesis was tested using hierarchical regression analysis (HRA) with an interaction term approach in SPSS. The results revealed a significant interaction effect (β = 0.17, *p* < 0.001), indicating that the influence of job crafting on organizational commitment becomes more pronounced among employees in AI departments as psychological ownership increases.

**Figure 3:**
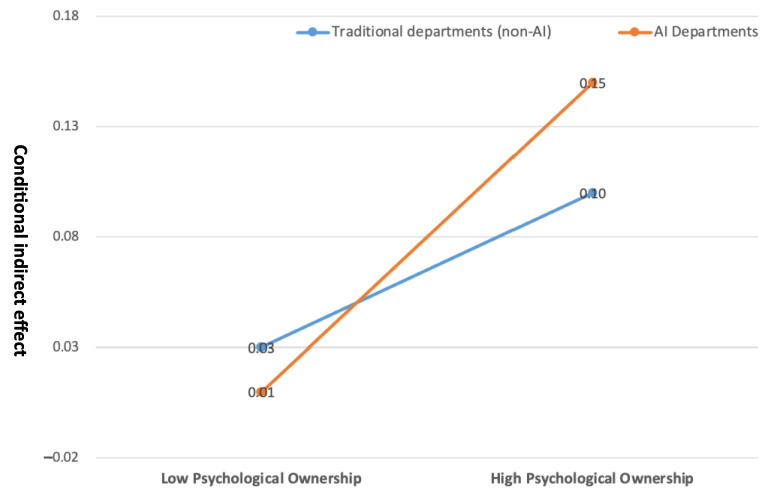
Comparative analysis of psychological ownership between AI and non-AI departments.

Figure 3 visually illustrates this interaction by comparing outcomes across AI and traditional (non-AI) departments at varying levels of psychological ownership. Psychological ownership was categorized into low and high levels, while department type (AI vs. traditional) served as the second factor. The plotted lines, based on mean values from the analysis, show a clear divergence: at low levels of psychological ownership, the outcome score for the AI department (0.01) was slightly lower than that of the traditional department (0.03). However, at high levels of psychological ownership, the score for the AI department increased markedly to 0.15, surpassing the traditional department’s score of 0.10. These findings suggest that psychological ownership exerts a stronger influence on organizational commitment in AI departments. Notably, employees in AI departments exhibited significantly higher levels of organizational commitment when psychological ownership was high, highlighting its greater motivational impact in technologically intensive environments. In sum, the results confirm that psychological ownership positively moderates the relationship between job crafting and organizational commitment, and that this moderating effect is stronger in AI departments. Hypothesis 2 is therefore supported.

## 6. Conclusions

### 6.1. Discussion

The key findings of this study are summarized as follows: First, organizational commitment mediates the relationship between job crafting and innovative behavior. This study provides a novel perspective on how job crafting influences innovation, addressing gaps in the existing literature by demonstrating that job crafting impacts innovative behavior through organizational commitment. Second, psychological ownership moderates the relationship between job crafting and organizational commitment. Employees in AI and non-AI departments exhibit significant differences in their sense of psychological ownership. Those with higher psychological ownership tend to be more engaged and responsible in their roles, which facilitates more effective job crafting and strengthens organizational commitment. Unlike previous studies that primarily focused on the direct effects of job crafting, this study emphasizes the moderating role of psychological ownership, offering a fresh theoretical perspective. Furthermore, as psychological ownership increases, the impact of job crafting on organizational commitment becomes more pronounced. Employees in AI departments generally have a stronger sense of ownership due to their direct involvement in developing and applying new technologies, thus extending the current theoretical understanding of psychological ownership. Third, the role of organizational commitment in linking job crafting and innovative behavior is further influenced by psychological ownership. Specifically, psychological ownership enhances this mediating relationship. Employees who experience a stronger sense of psychological ownership are more likely to exhibit higher levels of innovative behavior through job crafting, with organizational commitment serving as the mediator. These findings underscore the crucial role of psychological ownership in shaping the relationship between job crafting, organizational commitment, and innovative behavior, providing valuable insights for fostering employee-driven innovation.

This study, through a comparison of existing theoretical frameworks and empirical findings, reveals the unique role of PO in technology-driven organizations, offering a new theoretical perspective for organizational behavior research. Traditional studies, grounded in psychological ownership theory, often emphasize the linear strengthening effect of employees’ emotional attachment to their organization or tasks on their behavioral motivation (X. Zhao et al., 2022). These studies generally assume the universality of this mechanism across different work contexts. However, this study finds that the moderating effect of psychological ownership varies significantly between AI and non-AI departments, challenging the “homogenization” assumption in existing theories about the situational effects of moderating variables. For instance, job crafting theory, proposed by Wrzesniewski and Dutton (2001), suggests that employees enhance autonomy by adjusting task boundaries, which in turn boosts organizational commitment, but does not explain how the depth of technological embedding influences this process. This study shows that in AI departments, psychological ownership is more reflected in “creative control” over technological outcomes rather than traditional organizational attachment. When employees frequently participate in algorithm iterations or human–machine collaborations, the interaction between psychological ownership and job crafting further strengthens the “technological empowerment” aspect of innovative behavior. Building on Z. Zhang et al.’s (2025) “Technological Contextualized Moderation Theory”, this study is the first to empirically validate the influence of technological attributes on psychological mechanisms through cross-departmental comparisons. Although the existing literature often assumes a monotonic strengthening effect of psychological ownership on innovative behavior, this study uncovered a threshold effect using the Johnson–Neyman technique. Specifically, the moderating effect of psychological ownership on the relationship between job crafting and organizational commitment was significant only when psychological ownership was below a critical value—0.61 standard deviations below the mean. Beyond this threshold, the moderating effect diminished and became statistically non-significant. This finding highlights the nonlinear nature of psychological ownership’s moderating role, diverging from the linear enhancement assumptions commonly found in conventional models.

The results suggest that lower levels of psychological ownership may trigger a compensatory behavioral strategy, whereby employees, lacking a strong sense of belonging or control, proactively engage in job crafting to bridge psychological gaps and reestablish self-worth and role identity. However, this compensatory mechanism is not infinitely reinforcing. Instead, it follows a threshold pattern: once psychological ownership surpasses a certain level, its capacity to moderate the job crafting–organizational commitment relationship diminishes and may eventually become negligible. This insight offers a fresh perspective on the phenomenon of innovation fatigue among employees in high-demand, technology-intensive roles. It also challenges the managerial assumption that “more psychological ownership is always better”. Future research should further investigate the dynamic interactions between psychological ownership and contextual variables such as technological autonomy and task complexity, particularly in the era of widespread generative artificial intelligence. As employees increasingly co-create value alongside intelligent systems, the meaning of psychological ownership may shift—from a sense of organizational belonging to a notion of shared ownership over technological outcomes—necessitating theoretical reconceptualization and deeper empirical inquiry.

These findings have dual implications for management practice: First, managers in AI departments should strengthen employees’ sense of control over technological outcomes through technological empowerment (e.g., granting access to algorithm parameter adjustments), thereby activating the moderating potential of psychological ownership. Second, managers in non-AI departments should focus on building transparent role awareness and collaborative networks to help employees use job crafting to link their personal contributions with organizational strategies. This differentiated strategy breaks away from the traditional “one-size-fits-all” management approach, providing a theoretical anchor for organizational design in the AI era.

### 6.2. Theoretical Implications

This study makes several theoretical contributions to the empirical research on job crafting, organizational commitment, psychological ownership, and innovative behavior. First, it reinforces the critical role of organizational commitment as a mediator between job crafting and innovative behavior. This finding suggests that, in addition to direct job design strategies, fostering a strong sense of organizational commitment is essential for organizations seeking to stimulate innovation. Second, by highlighting the moderating role of psychological ownership in the relationship between job crafting and organizational commitment, this study emphasizes that psychological ownership is a key psychological factor that strengthens employee commitment. This moderating role provides new insights into how employees’ subjective sense of ownership over their roles can influence the path from job crafting to innovation. Third, this study identifies significant differences in psychological ownership between employees in AI and non-AI departments, offering valuable theoretical insights into how different job contexts shape employees’ psychological experiences and their impact on organizational commitment and innovative behavior. This finding extends the theoretical understanding of psychological ownership by showing how involvement in high-innovation, high-autonomy roles can amplify feelings of ownership, thereby deepening organizational commitment. Additionally, the differentiation in psychological ownership between AI and non-AI employees introduces a new perspective on how roles tied to technological advancement can intrinsically motivate employees to innovate. This study broadens the literature on psychological ownership by emphasizing that not all job roles foster ownership to the same degree. The findings offer a nuanced view of how psychological ownership functions across different departmental roles, highlighting the need for further exploration of how organizations can leverage job characteristics to cultivate a sense of ownership. In doing so, this study enriches the theoretical discourse on the interplay between psychological ownership, job context, and employee-driven innovation in the workplace.

### 6.3. Practical Implications

As knowledge-based economies advance and technology evolves, organizations face growing risks and competitive pressures, positioning employees as key drivers of innovation (Tang et al., 2021). To harness this potential, managers in Internet companies should foster an environment that encourages proactive job crafting. This includes revisiting performance evaluation and reward systems to promote individual innovative behavior and organizational commitment. Furthermore, leaders should facilitate open communication and collaboration, creating opportunities for employees to propose and implement innovative ideas. Adopting leadership styles that enhance participation and considering individual psychological factors through policies and programs are also crucial (Eva et al., 2020). For example, training programs designed to enhance autonomy and responsibility would be beneficial. Increasing transparency in role distribution and task organization within teams, while promoting idea sharing and cooperation, can help cultivate an environment where innovation is encouraged. These efforts also ensure that individuals receive appropriate rewards and recognition, strengthening their commitment to the organization. By balancing individual autonomy with alignment to collective goals, organizations can foster a culture of mutual respect and collaboration (Van den Hout & Davis, 2022). This collaborative culture is particularly important when considering the varying levels of psychological ownership between AI and non-AI departments, as these differences can significantly impact how effectively AI technologies are integrated for sustainable development. Additionally, implementing effective talent management strategies is essential for attracting, retaining, and developing professionals with both technical and non-technical expertise, ensuring the long-term success of AI-driven initiatives (Kulkov et al., 2024).

### 6.4. Limitations and Future Directions

Although this study provides valuable insights, several limitations warrant further exploration. First, while this study offers a comparative analysis of psychological ownership between AI and non-AI departments, the influence of AI extends beyond individual psychological factors to broader social dynamics. Future studies could explore different facets of the AI industry, such as its impact on team interactions, organizational culture, and social relationships. For instance, researchers might investigate the ethical considerations of AI adoption, including issues like fairness, accountability, and transparency in AI-driven decision-making (Kulkov et al., 2024). Second, this study primarily focuses on internal organizational factors, overlooking the influence of external environmental variables. To gain a more comprehensive understanding of innovation dynamics, future research should examine how external factors—such as competitive pressures and industry shifts—interact with internal elements to shape innovative behaviors. Additionally, further studies should address the challenges faced by mature companies adopting AI for sustainability, exploring strategies for digital transformation, operational restructuring, and the development of AI-driven business models for sustainable growth (Kulkov et al., 2024). Third, this study uses cross-sectional data, which limits the ability to establish definitive causal relationships among the variables. Chambel and Carvalho (2022) suggested that an individual’s psychological state, when sustained over time, could significantly impact their behavior. Therefore, future research could measure job crafting, organizational commitment, and psychological ownership at an initial time point, followed by an assessment of innovative behavior after a certain period.

## Figures and Tables

**Table 1:** Sample characteristics.

Variable		N = 457	%
Gender	Males	228	49.9
	Females	229	50.1
Age	≤25 years old	28	6.1
	25–35 (inclusive) years old	42	9.2
	35–45 (inclusive) years old	276	60.4
	45–55 (inclusive) years old	54	11.8
	>55 years old	57	12.5
Educational Background	College degree	63	13.8
	Bachelor’s degree	220	48.1
	Master’s degree	107	23.4
	Doctoral degree	67	14.7
Department	Traditional departments (non-AI)	314	68.7
	AI departments	143	31.3
Job Position	Staff Member	61	13.3
	Team leader	170	37.2
	Supervisor	172	37.6
	Manager	40	8.8
	Director and above	14	3.1
Years of Work Experience	≤1 year	12	2.6
	1–3 (included) years	38	8.3
	3–5 (included) years	17	3.7
	5–10 (included) years	182	39.8
	>10 years	208	45.5

**Table 2:** Fit indices of the research model.

CMIN	DF	χ^2^/df	CFI	TLI	IFI	RMSEA	SRMR
926.359	854	1.09	0.99	0.99	0.99	0.02	0.03

Note: χ^2^/df represents the ratio of the chi-square value to the degrees of freedom, commonly used to assess model fit, with a value below 3 indicating good model adequacy.

**Table 3:** Correlations analysis.

Variables	Mean	SD	1	2	3	4	5	6	7	8	9	10
1. Gender	1.50	0.50										
2. Age	2.85	0.97	0.02									
3. Educational Background	2.39	0.90	−0.05	−0.90 **								
4. Department	4.12	1.85	−0.06	−0.09	0.07							
5. Job Position	2.51	0.94	−0.08	−0.70 **	0.78 **	0.07						
6. Years of Work Experience	4.17	1.01	−0.04	−0.78 **	0.77 **	0.05	0.72 **					
7. Job Crafting	3.26	0.91	−0.00	−0.09	0.07	0.092 *	0.09	0.05	*0.59*(0.96)			
8. Organizational Commitment	3.29	0.96	−0.14 **	−0.06	0.05	0.13 **	0.06	0.05	0.41 **	*0.63*(0.96)		
9. Psychological Ownership	3.33	0.94	−0.03	−0.07	0.07	0.14 **	0.09	0.09 *	0.44 **	0.46 **	*0.60*(0.91)	
10. Innovative Behavior	3.31	0.93	−0.08	−0.10 *	0.10	0.12 **	0.11 *	0.05	0.41 **	0.39 **	0.42 **	*0.59*(0.90)

Note: n = 457. CR in the diagonal brackets. Italicized text indicates the AVE. * *p* < 0.05; ** *p* < 0.01.

**Table 4:** Results of hierarchical regression analysis.

Variables	Organizational Commitment				Innovative Behavior		
	Model 1	Model 2	Model 3	Model 4	Model 5	Model 6	Model 7
Gender	−0.14	−0.14	−0.13	−0.12	−0.07	−0.07	−0.03
Age	−0.09	−0.02	−0.04	−0.05	−0.17	−0.11	−0.10
Educational Background	−0.07	−0.02	−0.01	−0.02	−0.11	−0.07	−0.06
Department	0.12	0.09	0.05	0.03	0.11	0.08	0.05
Job Position	0.04	−0.00	−0.00	0.00	0.14	0.10	0.10
Years of Work Experience	−0.01	0.02	−0.02	−0.03	−0.11	−0.08	−0.09
JC		0.40 ***	0.26 ***	0.22 ***		0.39 ***	0.27 ***
PO			0.33 ***	0.33 ***			
Int				0.17 ***			
OC							0.30 ***
F	3.07	15.89 ***	22.42 ***	22.46 ***	2.84	10.36 ***	12.53 ***
R^2^	0.04	0.20	0.29	0.31	0.04	0.18	0.25
∆R^2^	-	0.16	0.09	0.02	-	0.14	0.07
Mediated Effect Via Organizational Commitment				0.13(0.02) [0.09, 0.18]			
Moderated Mediation Effects of Psychological Ownership				0.06(0.02) [0.03, 0.10]			

Note: n = 457. JC: Job Crafting, PO: Psychological Ownership, OC: Organizational Commitment, Int = Job Crafting × Psychological Ownership. *** *p* < 0.001.

**Table 5:** Regression analysis of job crafting, psychological ownership, and their interaction on organizational commitment.

Variable	Coefficient	Standard Error	*p*-Value	Significance Interval
Job Crafting	0.22	0.05	<0.001	-
Psychological Ownership	0.32	0.05	<0.001	-
Interaction (JC:PO)	0.16	0.04	<0.001	[−3.80, −0.61]

## Data Availability

The raw data supporting the conclusions of this article will be made available by the authors on request.

## Appendix A. Measurement Items

Job Crafting

1. Introduce new approaches to improve your work.
2. Change the scope or types of tasks that you complete at work.
3. Introduce new work tasks that you think better suit your skills or interests.
4. Choose to take on additional tasks at work.
5. Give preference to work tasks that suit your skills or interests.
6. Think about how your job gives your life purpose.
7. Remind yourself about the significance your work has for the success of the organization.
8. Remind yourself of the importance of your work for the broader community.
9. Think about the ways in which your work positively impacts your life.
10. Reflect on the role your job has for your overall well-being.
11. Make an effort to get to know people well at work.
12. Organize or attend work related social functions.
13. Organize special events in the workplace (e.g., celebrating a co-worker’s birthday).
14. Choose to mentor new employees (officially or unofficially).
15. Make friends with people at work who have similar skills or interests.

Organizational Commitment

1. I am willing to put in a great deal of effort beyond that which is normally expected to help this company be successful.
2. I talk up this company to my friends as a great company to work for.
3. I am proud to tell others that I am part of this organization.
4. IamextremelygladthatIchosethiscompanytoworkforoverwhatIwasconsidering at the time I joined.
5. I really care about the future of this company.
6. For me, this is the best of all possible organization for which to work.
7. I find that my values and the company’s values are similar.

Psychological Ownership

1. This is my organization.
2. I sense that this organization is our company.
3. I feel a very high degree of personal ownership for this organization.
4. I sense that this is my company.
5. This is our company.
6. Most of the people that work for this organization feel as though they own the company.
7. It is hard for me to think about this organization as mine.

Innovative Behavior

1. Searches out new technologies, processes, techniques, and/or product ideas.
2. Generates creative ideas.
3. Promotes and champions ideas to others.
4. Investigates and secures funds needed to implement new ideas.
5. Develops adequate plans and schedules for the implementation of new ideas.
6. Is innovative.
